# Supplementary material for: From sequence to enzyme mechanism using multi-label machine learning
Source: BMC Bioinformatics. 2014 May 19;15:150. doi: 10.1186/1471-2105-15-150 (PMC4229970; doi:10.1186/1471-2105-15-150)
Supplement: Additional file 2 — Java code of ml2db. Additional file ml2db_code.tar.gz contains the Java source code to run the multi-label machine learning experiments and save the results to database. The code’s Javadoc is included. [file 1471-2105-15-150-S2.zip › additional file 2/ml2db/utils/doc/uk/ac/ed/inf/utils/database/DbReader.html]

DbReader


---


|  |  |  |
| --- | --- | --- |
| **Overview** \| **Package** \| **Class** \| **Use** \| **Tree** \| **Deprecated** \| **Index** \| **Help** | |  |
| **PREV CLASS**   **NEXT CLASS** | **FRAMES**    **NO FRAMES**     **All Classes** |  |
| SUMMARY: NESTED \| FIELD \| CONSTR \| METHOD | DETAIL: FIELD \| CONSTR \| METHOD |  |


---


## uk.ac.ed.inf.utils.database Class DbReader<T,U>

```
java.lang.Object
  uk.ac.ed.inf.utils.database.DbManaged
      uk.ac.ed.inf.utils.database.DbReader<T,U>
```

---

``` public class DbReader<T,U> extends DbManaged ```

Class to read from a database. For queries: if a set of queries point
repeatedly to the same table, or you need table specific information, get a
`TableReader` object.

**Version:**
:   20 Feb 2009

**Author:**
:   Luna De Ferrari luna.deferrari-at-ed.ac.uk

---

**Constructor Summary**

`DbReader(DbManager dbManager)`


| **Method Summary** | |
| --- | --- |
| `java.sql.ResultSet` | `executeQuery(java.lang.String sql)`             Executes an sql query returning a result set. |
| `OneToManyMap<T,U>` | `get2ColumnsValues(java.sql.ResultSet resultSet, int columnIndexOfKeys, int columnIndexOfValues, OneToManyMap<T,U> map, int expectedNumberOfKeysUniqueStrings, int expectedNumberOfValuesUniqueStrings)`             Get the nth and mth column of a `ResultSet` |
| `java.util.AbstractCollection<T>` | `getColumnValues(java.sql.ResultSet resultSet, int columnIndex, java.util.AbstractCollection<T> collection)`             Get the nth column of a `ResultSet` |
| `java.sql.DatabaseMetaData` | `getDbMetaData()` |
| `java.util.TreeSet<java.lang.String>` | `getListOfDbTables()`             Gets the list of tables for a database |
| `java.util.TreeMap<java.lang.String,java.lang.String>` | `getMap(java.lang.String sql)`             Converts a query resultset into a map. |
| `OneToManyMap<T,U>` | `getMap(java.lang.String sql, int columnIndexOfKeys, int columnIndexOfValues, int expectedNumberOfKeysUniqueStrings, int expectedNumberOfValuesUniqueStrings)`             Converts a query resultset into a map. |
| `java.sql.Statement` | `getStatement()` |
| `TableReader` | `getTableReader(java.lang.String table)`             Object to read from a table |
| `OneToManyMap<T,U>` | `queryResultsColumnsToMap(java.lang.String sql, int columnIndexOfKeys, int columnIndexOfValues, int expectedNumberOfKeysUniqueStrings, int expectedNumberOfValuesUniqueStrings)`             From two resultset columns to a key-value map |
| `java.util.AbstractCollection<T>` | `queryResultsColumnToCollection(java.lang.String sql, int columnIndex, java.util.AbstractCollection<T> collection)` |
| `java.util.TreeSet<java.lang.String>` | `queryResultsColumnToSet(java.lang.String sql, int columnIndex)`             Reduces a query results column to a set (beware: it eliminates duplicates!) |
| `java.util.Vector<T>` | `queryResultsColumnToVector(java.lang.String sql, int columnIndex)` |
| `boolean` | `tableExistsInDb(java.lang.String tableName)`             Checks if a table exists in the database |

**Methods inherited from class uk.ac.ed.inf.utils.database.DbManaged**

`getDbConnection, getDbManager`

**Methods inherited from class java.lang.Object**

`equals, getClass, hashCode, notify, notifyAll, toString, wait, wait, wait`

**Constructor Detail**

### DbReader

```
public DbReader(DbManager dbManager)
```


**Method Detail**

### executeQuery

```
public java.sql.ResultSet executeQuery(java.lang.String sql)
```

:   Executes an sql query returning a result set.

    :   **Parameters:**: `sql` - an SQL query **Returns:**: a `ResultSet` with the query results **Throws:**: `java.sql.SQLException`

---


### getColumnValues

```
public java.util.AbstractCollection<T> getColumnValues(java.sql.ResultSet resultSet,
                                                       int columnIndex,
                                                       java.util.AbstractCollection<T> collection)
```

:   Get the nth column of a `ResultSet`

    :   **Parameters:**: `resultSet` -: `columnIndex` - the column number (starting at 0) **Returns:**: the column values

---


### get2ColumnsValues

```
public OneToManyMap<T,U> get2ColumnsValues(java.sql.ResultSet resultSet,
                                           int columnIndexOfKeys,
                                           int columnIndexOfValues,
                                           OneToManyMap<T,U> map,
                                           int expectedNumberOfKeysUniqueStrings,
                                           int expectedNumberOfValuesUniqueStrings)
```

:   Get the nth and mth column of a `ResultSet`

    :   **Parameters:**: `resultSet` -: `columnIndexOfKeys` - the nth column (columns index starting at 0): `columnIndexOfValues` - the mth column (columns index starting at 0): `map` - the map to put the keys and values in: `expectedNumberOfResultsetUniqueValues` - the expected number of \*unique\* string values in the resultset **Returns:**: a map containing the result set data as keys and values of a map

---


### getDbMetaData

```
public java.sql.DatabaseMetaData getDbMetaData()
```

---


### getListOfDbTables

```
public java.util.TreeSet<java.lang.String> getListOfDbTables()
```

:   Gets the list of tables for a database

    :   **Returns:**: a list of table names

---


### getMap

```
public java.util.TreeMap<java.lang.String,java.lang.String> getMap(java.lang.String sql)
```

:   Converts a query resultset into a map. Beware: Null values are discarded
    both as keys and as values.

    :   **Parameters:**: `sql` - the sql query (the first column should be the key and the second column the value) **Returns:**: the map with the resultset results

---


### getMap

```
public OneToManyMap<T,U> getMap(java.lang.String sql,
                                int columnIndexOfKeys,
                                int columnIndexOfValues,
                                int expectedNumberOfKeysUniqueStrings,
                                int expectedNumberOfValuesUniqueStrings)
```

:   Converts a query resultset into a map. Beware: Null values are discarded
    both as keys and as values.

    :   **Parameters:**: `sql` - the sql query: `columnIndexOfKeys` - the index for the results column which contains the keys (eg. 0): `columnIndexOfValues` - the index for the results column which contains the values (eg. 1) **Returns:**: the map with the resultset results

---


### getStatement

```
public java.sql.Statement getStatement()
```

---


### getTableReader

```
public TableReader getTableReader(java.lang.String table)
```

:   Object to read from a table

---


### queryResultsColumnsToMap

```
public OneToManyMap<T,U> queryResultsColumnsToMap(java.lang.String sql,
                                                  int columnIndexOfKeys,
                                                  int columnIndexOfValues,
                                                  int expectedNumberOfKeysUniqueStrings,
                                                  int expectedNumberOfValuesUniqueStrings)
```

:   From two resultset columns to a key-value map

    :   **Parameters:**: `sql` -: `columnIndexOfKeys` -: `columnIndexOfValues` -: `map` -: `expectedNumberOfKeysUniqueStrings` -: `expectedNumberOfValuesUniqueStrings` - **Returns:**: a map with two of the resultset columns as keys and values respectively

---


### queryResultsColumnToCollection

```
public java.util.AbstractCollection<T> queryResultsColumnToCollection(java.lang.String sql,
                                                                      int columnIndex,
                                                                      java.util.AbstractCollection<T> collection)
```

---


### queryResultsColumnToSet

```
public java.util.TreeSet<java.lang.String> queryResultsColumnToSet(java.lang.String sql,
                                                                   int columnIndex)
```

:   Reduces a query results column to a set (beware: it eliminates
    duplicates!)

    :   **Parameters:**: `sql` - a query to extract the columns from database: `columnIndex` - the index of the column of interest **Returns:**: the set of column results

---


### queryResultsColumnToVector

```
public java.util.Vector<T> queryResultsColumnToVector(java.lang.String sql,
                                                      int columnIndex)
```

---


### tableExistsInDb

```
public boolean tableExistsInDb(java.lang.String tableName)
```

:   Checks if a table exists in the database

    :   **Parameters:**: `tableName` - **Returns:**: true if a table with the given name exists in the database


---


|  |  |  |
| --- | --- | --- |
| **Overview** \| **Package** \| **Class** \| **Use** \| **Tree** \| **Deprecated** \| **Index** \| **Help** | |  |
| **PREV CLASS**   **NEXT CLASS** | **FRAMES**    **NO FRAMES**     **All Classes** |  |
| SUMMARY: NESTED \| FIELD \| CONSTR \| METHOD | DETAIL: FIELD \| CONSTR \| METHOD |  |


---
